# Understanding the unique flowering sequence in *Dipsacus fullonum*: Evidence from geometrical changes during head development

**DOI:** 10.1371/journal.pone.0174091

**Published:** 2017-03-22

**Authors:** Somayeh Naghiloo, Regine Claßen-Bockhoff

**Affiliations:** Instituts für Organismische und Molekulare Evolutionsbiologie, Johannes Gutenberg-Universität, Mainz, Germany; Nanjing Agricultural University, CHINA

## Abstract

The genus *Dipsacus* is characterized by a remarkable bidirectional flowering sequence and a rare phyllotactic pattern. Considering that flower initiation and flowering sequence may be interconnected, we document the development of the head meristem in *Dipsacus fullonum*. Our results indicate a gradual change in the geometry of the head meristem beginning with a dome shaped stage, continuing with a remarkable widening in the middle part of the head meristem and ending in a spindle-like form. Quantitative data confirm that meristem expansion is higher in the middle part than at the base of the meristem. Likewise, the size of the flower primordia in the middle part of the young head is significantly larger than at the base soon after initiation. We conclude that the change in the geometry of the meristem and the availability of newly generated space result in the promotion of the middle flowers and the bidirectional flowering sequence at anthesis. Our investigation on phyllotactic patterns reveals a high tendency (30%) of the head meristem to insert or lose parastichies. This finding can also be attributed to changes in the expansion rate of the meristem. Dependent on the spatio-temporal relation between meristem expansion and primordia initiation, either flower primordia are promoted or additional parastichies appear. Our results emphasize the important role of geometry in flower development and phyllotactic pattern formation.

## Introduction

**Fig 1:**
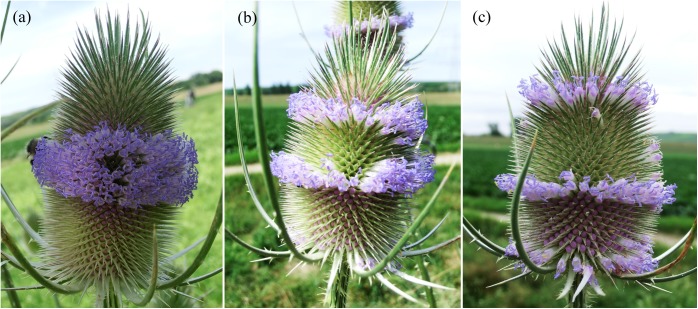
Bidirectional flowering in the head of *Dipsacus fullonum*. (a) The middle flowers start flowering. (b, c) Flowering sequence proceeds both upward and downward

The wild teasel, *Dipsacus fullonum* L., is a perennial to biennial herb of the family Caprifoliaceae-Dipsocoideae [1]. The genus is mainly recognized by the presence of stem prickles, egg-shaped flower-heads and spine-tipped bracts. It has long been characterized by its remarkable pattern of flowering [2]. Ordinarily, one would expect that flowering begins at the base and extends towards the tip, but in *Dipsacus* it begins with the middle flowers and then extends both upward and downward. This gives the impression of two rings of open flowers which ‘migrate’ towards the poles of the head over subsequent days (Fig 1). How this interesting flowering sequence is actually originated is still an unsolved puzzle. The enigma of the flowering pattern in *Dipsacus* is paralleled by the special phyllotaxis of the head and irregularities in parastichies numbers [3].

The flowering sequence plays a determinant role in the reproductive success of a plant due to its strong effects on pollination and fruit set. Within a plant, the flowering sequence is highly diverse, e.g. acropetal, basipetal or simultaneously, depending on the branching system [4]. Within an inflorescence, it is usually constant and originates during early stages of development. Each type of inflorescence is characterized by a special developmental pattern which results in a specific flowering sequence. In racemose inflorescences, the tipward growth of the meristem results in an acropetal flowering sequence, and in thyrses, the cymes flower in an ordinal sequence following the branch orders. Head meristems are completely used by flower primordia, which originate and flower in a centripetal direction [4]. Rarely, the flowering sequence is almost simultaneous (e.g. *Mimosa pudica*) or basipetal (e.g. *Sanguisorba minor*; secondary heads in *Echinops* spp.) [4]. The bidirectional flowering sequence in *Dipsacus* represents an exceptional case. Beside internal developmental factors, external factors such as changes in light intensity and relative humidity can regulate flower opening and flowering sequence [5]. As developmental studies in *Dipsacus fullonum* are lacking, it is not clear whether the unusual flowering sequence is a late developmental effect, which is regulated by external factors, or originates early in development.

The surprising tendency of plant organs to occur preferentially in some specific numbers has attracted a stream of mathematicians, computer scientists and physicists along with botanists and plant biologists. According to dynamic models of phyllotaxis, primordia formation is a self-regulating process providing optimal packing and filling space completely [6–10]. During organogenesis, the expansion of the meristematic surface changes the density and proximity of flowers and new primordia are usually inserted where space is available [6, 10, 11]. In this view, the geometry of the apex during development could have a potential role in the organization of inflorescence structure and phyllotactic patterns [12]. Though phyllotaxis often shows a surprising mathematical regularity, observations also indicate a high degree of variability and disorder [13, 14]. Understanding the mechanistic base of such disorders will improve our knowledge about the robustness of phyllotactic patterns.

We hypothesize that the unusual flowering sequence and parastichy distortion in *Dipsacus fullonum* are most probably due to changes in spatial conditions during meristem expansion influencing primordia formation. In order to confirm this hypothesis, we study the development of the heads in *Dipsacus fullonum*. The main questions are (1) at which developmental stage the irregular flowering sequence appear, (2) to which extent phyllotactic distortions occur in the head, (3) how the shape of the meristem changes during development, and (4) how far the irregular flowering sequence and parastichies are correlated with geometric changes in the head meristem.

## Materials and methods

### Scanning electron microscopy (SEM)

Head buds of *Dipsacus fullonum* L. were collected in June 2015 at the Botanical Garden of the Johannes Gutenberg-Universität Mainz (Germany). Plant material was fixed in 70% EtOH and then dehydrated in an alcohol–acetone series. The samples were critically point-dried (BAL-TEC CPD030), mounted and sputter coated with gold (BAL-TEC SCD005) and observed under the scanning electron microscope (ESEM XL-30 Philips). All steps were conducted according to the manufacturer’s protocols.

### Morphometry

To quantify meristem expansion during development, 30 heads were randomly chosen from five individuals and classified into three developmental stages: (S1) dome-shaped meristem in which the primordia start to appear in parastichies, (S2) meristem with flower primordia covering more than half of the meristem height, (S3) meristem at the end of flower initiation, before the onset of floral organ formation. The diameters at the basal and middle parts were measured in each different developmental stage (10 heads per developmental stage). To correlate the meristem expansion in S3 with the promotion of the middle flowers, the average head diameter and the average extent of flower meristems (10 heads and four flowers per head per zone) were measured at the bottom, middle and top of the head.

All measurements were made using digimizer software (http://www.download.hr/download-digimizer.html). Statistical analyses were carried out by single factor analysis of variance (ANOVA). Results were considered significant for p<0.05.

### Analysis of phyllotactic patterns

To study the regularity of the phyllotactic pattern, 40 developed heads from 10 individuals were collected. The number of parastichies was counted in clockwise and counter clockwise directions starting from the bottom of the head. Detailed observation along entire parastichy lines was carried out in order to detect any kind of distortion in the form of adding or loosing parastichies.

## Results

### Head development (Fig 2)

**Fig 2:**
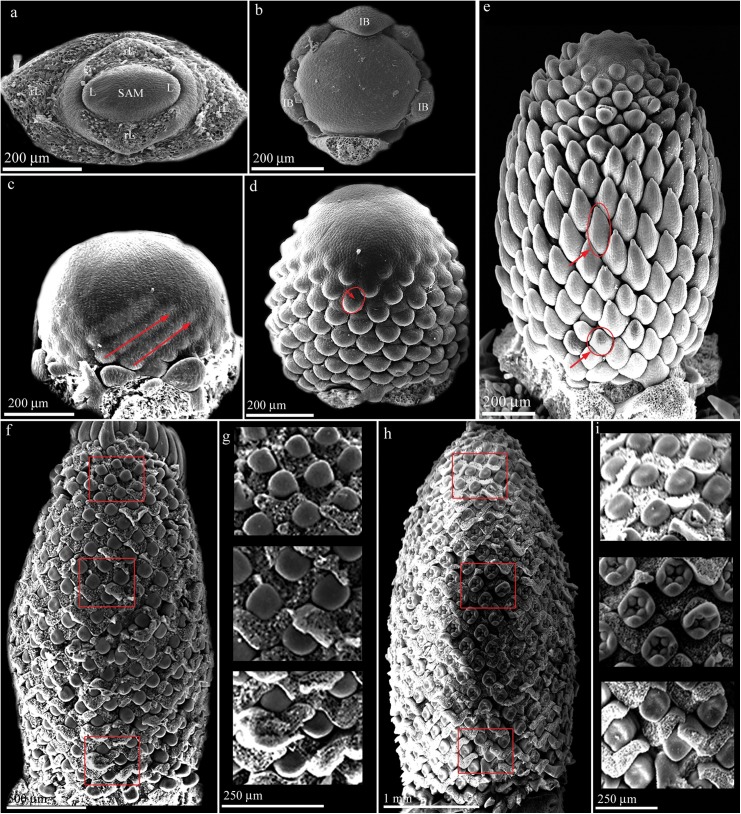
Head development in *Dipsacus fullonum*. (a) Vegetative shoot apical meristem (SAM). (b) Naked head meristem. (c) First flowers are initiated in contact parastichies; some parastichies are delayed compared to adjacent ones (arrows). (d) Elongation and expansion of the head meristem and acropetal formation of parastichies. (e) Completion of floral initiation. Note the spindle-like shape of the meristem and the smaller size of the basal bracts (arrow) compared to the middle ones (arrow). (f) Mature head with all bracts removed except at the top. (g) Details from (f) showing the larger size of the middle flower primordia compared to the basal and apical ones (same scale). (h) Flower organogenesis starts in the middle flowers and proceeds towards both ends. (i) Details from (h) showing the promotion of the development in the middle flowers (same scale). IB = involucral bracts; L = leaf; rL = removed leaf; SAM = shoot apical meristem.

The relatively small vegetative shoot apical meristem (SAM) produces decussately arranged leaves (Fig 2A: L), enlarges and changes into a large naked meristem (Fig 2B). This meristem produces a set of involucral bracts (Fig 2B: IB). The meristem becomes globular and the first primordia appear in parastichies (Fig 2C). The primordia are roundish and later split into bracts and flowers (Fig 2D: arrow). Flower initiation proceeds in an acropetal way with spiral parastichies (Fig 2C). In some parastichies (Fig 2C: arrows), the flower initiation is delayed compared to adjacent parastichies. As growth continues, the young head attains a more or less spindle-like shape with considerable radial expansion in the middle part (Fig 2D and 2E). Further initiation of bracts and flowers continues acropetally, filling the remaining meristematic space (Fig 2E). Concurrently with the completion of flower initiation, the bracts grow more and cover the flower primordia (Fig 2E). At this stage, the bracts at the middle zone of the head are larger than the bracts at the base (Fig 2E: arrows). After their removal, it is obvious that the middle (younger) flower primordia are promoted compared to the basal (older) flower primordia (Fig 2F and 2G). Organ initiation starts first in the middle flowers and then proceeds acropetally and basipetally (Fig 2H and 2I). This promotion is retained throughout development until anthesis finally resulting in the bidirectional flowering sequence.

### Quantitative analysis of meristem expansion and flower growth (Fig 3, S1 and S2 Tables)

**Fig 3:**
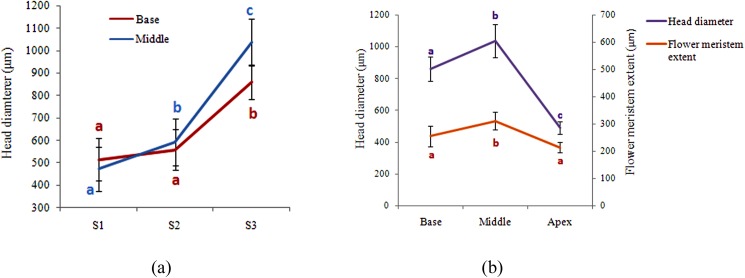
Quantitative changes during head development. (a) Head diameter at the basal and middle part in three developmental stages (S1, S2, S3); meristem expansion starts earlier in the middle part and shows a higher rate compared to the basal part. (b) Comparison of head diameter and flower primoridum size in the basal, middle and apical zones of the heads in stage S3. The expansion of the head in the middle part is paralleled with the promotion of the middle flowers (n = 10 per developmental stage). For each measured trait, characters that share the same letter do not differ significantly on the basis of Tukey’s test (p<0.05).

To quantify the expansion of the head meristem during development, the diameter of the head in the basal and middle areas was compared at three developmental stages (S1, S2 and S3) (Fig 3A). In S1 and S2, there is no significant difference between the zones. While the diameter of the head in the middle part significantly increases from S1 (472±97 μm in) to S2 (593± 105 μm), no significant difference in the diameter of the head bases is detectable (S1: 515± 96 μm; S2: 558±89 μm). In S3, the diameter of both meristem zones significantly increases compared to S2. This increase is more prominent in the middle part (from 593±105 μm to 1036±105 μm) than at the base (from 558±89 μm to 859±77 μm). Altogether, meristem expansion starts earlier and with a higher rate in the middle zone of the head meristem than at its base.

In order to correlate the changes in space and flower size, the head diameter and the average flower primordium size were measured in S3 for each of the three zones (base, middle, top) of the head (Fig 3B). The average diameter in the middle part of the head is with 1036±105 μm significantly larger than in its basal (859±77 μm) and apical parts (489±40 μm). Interestingly, the average flower primordium size is likewise the highest in the middle part of the head (311±32 μm) followed by the basal (255±38 μm) and apical parts (214±20 μm). The oldest flowers at the base of the head are thus significantly smaller than the middle ones which are later initiated. The results reveal a highly significant positive correlation between flower size and head diameter at the basal, middle and apical zone of the head (r^2^ = 0.56, P< 0.0001).

### Analysis of phyllotactic pattern (Fig 4, Table 1)

**Fig 4:**
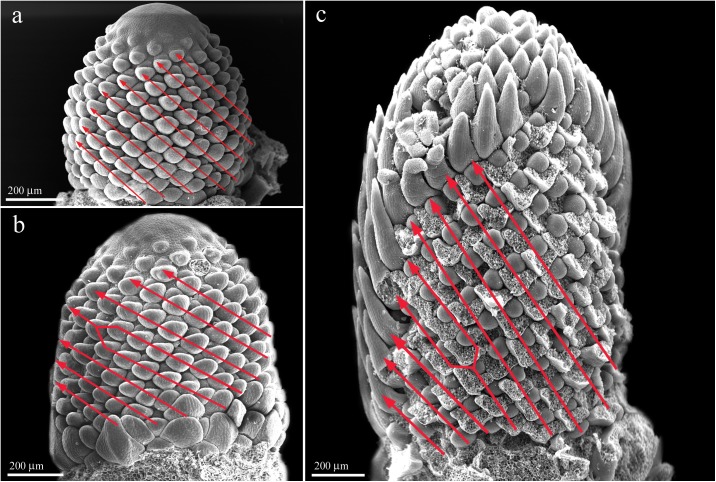
Parastichy formation in *Dipsacus fullonum*. (a) Regular pattern. (b) Parastichies unite at the tip of the meristem. (c) Additional contact lines appear after meristem expansion.

The majority of the heads show a regular formation of parastichies (Fig 4A), but distortions due to losing or adding parastichies are also observed (Fig 4B and 4C).

**Table 1:** Phyllotactic patterns in *Dipsacus fullonum* (n = 40).

Phyllotactic pattern	Number of heads	Percentage of heads	Number of heads with parastichy distortions*
**Bijugate series**	**27**	67.5%	5
16:26			
**Multijugate series**	**4**	10%	4
20:32	3		
25:40	1		
**Unusual series**	**3**	7.5%	3
19:24	1		
14:23	1		
28:48	1		
**Irregular series (without detectable spirals)**	**6**	15%	-

*Distortion in the form of additional or lacking parastichies.

Within the 40 analyzed heads of *Dipsacus*, six phyllotactic series are found in 34 heads (Table 1). The majority of the parastichy pair numbers belongs to the bijugate series (67.5%) which is followed by a multiplicity of Fibonacci numbers (10%). Multijugate series are based on `n´ Fibonacci numbers (i.e., 1, 1, 2, 3, 5, 8, …) with `n´ being 2 (bijugate; 2, 2, 4, 6, 10, 16,…), 3 (trijugate; 3, 3, 6, 9, 15, 24, …) or more. The phyllotaxis of the six remaining heads is so irregular that it cannot be classified. We observe unusual parastichy pair numbers (19:24, 14:23, 28:48) which do not fit into any reported phyllotactic pattern. Distortion of parastichies in the form of adding or loosing lines occurs in 30% of the analyzed heads, changing the original parastichy number. Such distortions are detected in all multijugate and unusual series and in some bijugate heads.

## Discussion

### Change in meristem geometry and unique flowering sequence

The results of our ontogenetic study in *Dipsacus fullonum* indicate that the unusual flowering sequence is not influenced by external factors. The promotion of flowers in the middle part of the head starts rather early in development and spatial constrains play an important role in the remarkable divergent flowering sequence.

**Fig 5:**
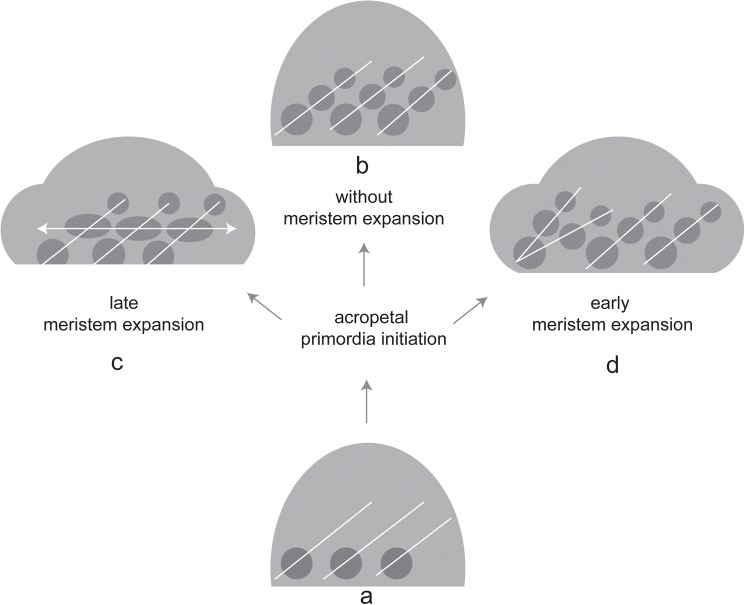
Schematic diagram illustrating the putative effects of early and late meristem expansion on flower initiation. (a) The head meristem fractionates flower primordia in an acropetal direction (white lines). (b) If the geometry of the head does not change, regular parastichies appear. (c, d) If the meristem expands in its middle part, spatial conditions change. Depending on the time of expansion relative to the time of primordia initiation, already existing primordia are promoted (c) or additional parastichies are formed (d).

In *Dipsacus fullonum*, the head geometry changes gradually during development (Fig 5) starting with a globular meristem and ending in a formation of a spindle-shaped head which is thicker in the middle than at both ends. The remarkable widening of the meristem in its middle part may be based on increased cell division and/or cell elongation. It generates new space which goes along with the promotion of flowers in this area. The proximal primordia, which are initiated first, become relatively smaller than the middle flowers. The growth precedence of the middle flowers is maintained throughout the development and reflected in the bidirectional flowering sequence at anthesis.

According to our quantitative study, each part of the head has its particular growth rate. A similar form of partitioning in the development of the inflorescence has been reported in *Philodendron* (Araceae) [15]. *Philodendron* has spadix inflorescences with female flowers at the base, followed by hermaphrodite and male flowers in the middle and apical zones. Based on a quantitative analysis, the authors showed that the growth rate is different in each of the three inflorescence zones which can be associated with the changes in flower development [15]. Our results also confirm the important role of geometry and growth dynamic in flower development.

In inflorescences, the flowers usually open according to their initiation sequence, i.e. acropetally in racemes, ordinally in cymes and centripetally in heads [4]. While the primordia appear acropetally in *Dipsacus*, their size changes after initiation which results in a bidirectional flowering at anthesis. Further examples are known with a divergence between initiation and flowering sequence.

- In some Asteraceae, delayed formation of ray flowers produces a bidirectional initiation sequence [16, 17]. However, the ray flowers expand shortly before anthesis and overtake the disk flowers in length which result in a centripetal flowering sequence.
- A bidirectional initiation sequence is also detected in *Davidia involucrata* (Cornaceae) which is followed by simultaneous flowering at anthesis [18]. The delayed formation of peripheral flowers in *Davidia* is shown to be related to the basal expansion of the meristem and generation of new space [18].
- In *Sanguisorba minor* (Rosaceae), initiation and later development of the flowers always proceed in an acropetal order. However, the female flowers, which are the youngest ones on the top of the head, open the first producing a basipetal flowering sequence [4]. We assume that hormones influence the flowering sequence in *Sanguisorba* resulting in protogyny.

Taken all examples together, the initiation of primordia, their development, and the opening of the flowers are not strictly interconnected. They refer to different levels of regulation which are influenced by mechanical (availability of space) and chemical (hormone distribution) changes.

### Change in geometry of apex and distortion of the parastichies

In *Dipsacus* the diversity of phyllotactic series is high, in which the Fibonacci series are replaced by bijugate or multijugate series. Our results are in agreement with Church [3] who reported the prevalence of bijugate series in *Dipsacus*. While the Fibonacci pattern is dominant in head of Asteraceae and cones of pineapple [3, 19], departures from Fibonacci pattern have been reported in gynoecia of *Magnolia acuminata* [20], vegetative shoots of some gymnosperms such as *Torreya* or *Cephalotaxus* [21] and microphylls of *Lycopodium* [22]. In *Lycopodium*, it is suggested that the change in the apex symmetry is the main factor contributing to the high phyllotactic diversity [22]. Recent studies revealed a correlation between the meristem size and the robustness of phyllotaxis in *Arabidopsis* [23]. These examples suggest an important role for meristem size and geometry in the establishment of phyllotactic pattern. Beside internal genetic factors, the meristem size can be influenced by environmental factors such as changes in day length [23, 24].

The relatively high tendency for phyllotactic distortion and irregularities in *Dipsacus* can be explained by the dynamic changes in meristem expansion. We postulate that additional parastichies result from meristem expansion in the middle part generating new space for additional primordia (Fig 5). On the other hand, a dramatic decrease in the rate of meristem expansion and consequently the head diameter in the apical part can explain the suppression of parastichy lines. The appearance of phyllotactic distortion appears to depend on the spatio-temporal relation between meristem expansion and primordia initiation. Hypothetically, if meristem expansion occurs after the establishment of the phyllotactic pattern, flower primordia may be promoted (Fig 5C), and if it occurs before the establishment of the phyllotactic pattern, additional parastichies may be formed (Fig 5D).

Our results indicate that growth rate and allometry of the meristem during development play an important role in the establishment of high-order phyllotactic patterns and subtle changes in the dynamics of meristematic growth versus primordia initiation can lead to irregularities and distortion of common patterns.

## Conclusion

The present study indicates a change in the geometry of the head meristem during development influencing flowering sequence and phyllotactic pattern. Given the impact of growth processes on flower development, integrating developmental studies on inflorescence and flowers will provide a better understanding of the developmental constrains of flowering.

According to our results, the geometrical analysis of the meristem during development is an important step towards constructing realistic models of the mechanisms responsible for generating phyllotactic patterns.

## Supporting information

S1 Table Comparison of head diameter and flower meristem extent in the basal, middle and apical zones of the heads in stage S3 (Mean values and standard deviation from 10 measurements). (DOC)

S2 Table Head diameter at the basal and middle parts in three developmental stages (S1, S2, S3). Mean values and standard deviation from 10 measurements.
(DOC)
