# Correction: Understanding the unique flowering sequence in *Dipsacus fullonum*: Evidence from geometrical changes during head development

**DOI:** 10.1371/journal.pone.0178582

**Published:** 2017-05-23

**Authors:** Somayeh Naghiloo, Regine Claßen-Bockhoff

The author Regine Claßen-Bockhoff should be listed as a second Corresponding Author for this article. The contact information for this author is classenb@uni-mainz.de.
