# Supplementary material for: Understanding the unique flowering sequence in Dipsacus fullonum: Evidence from geometrical changes during head development
Source: PLoS One. 2017 Mar 22;12(3):e0174091. doi: 10.1371/journal.pone.0174091 (PMC5362205; doi:10.1371/journal.pone.0174091)
Supplement: S1 Table — (DOC) [file pone.0174091.s001.doc]

**S1 Table**: Comparison of head diameter and flower meristem extent in the basal, middle and apical zones of the heads in stage S3 (Mean values and standard deviation from 10 measurements).

|  | Base | Middle | Apex |
| --- | --- | --- | --- |
| Head diameter (μm) | 844,9±80 | 1029,2±121 | 480,7±42 |
| Flower meristem extent (μm) | 255,5±38,0 | 311,2±32,1 | 214,3±20,0 |
