# Supplementary material for: Understanding the unique flowering sequence in Dipsacus fullonum: Evidence from geometrical changes during head development
Source: PLoS One. 2017 Mar 22;12(3):e0174091. doi: 10.1371/journal.pone.0174091 (PMC5362205; doi:10.1371/journal.pone.0174091)
Supplement: S2 Table — Mean values and standard deviation from 10 measurements. (DOC) [file pone.0174091.s002.doc]

**S2 Table**: Head diameter at the basal and middle parts in three developmental stages (S1, S2, S3). Mean values and standard deviation from 10 measurements.

|  | Base | Middle |
| --- | --- | --- |
| S1 | 515,0±96 | 472,5±97 |
| S2 | 558,5±89 | 593,0±105 |
| S3 | 859,5±77 | 1036,0±105 |
